# The Detailed Bactericidal Process of Ferric Oxide Nanoparticles on *E. coli*

**DOI:** 10.3390/molecules23030606

**Published:** 2018-03-08

**Authors:** Yunqiao Li, Dong Yang, Shang Wang, Chenyu Li, Bin Xue, Lin Yang, Zhiqiang Shen, Min Jin, Jingfeng Wang, Zhigang Qiu

**Affiliations:** 1 Department of Environment and Health, Tianjin Institute of Environmental and Operational Medicine, Tianjin 300050, China; liyunqiao_tj@126.com (Y.L.); yangd8611@163.com (D.Y.); wsh847@163.com (S.W.); nk_lcy710430@hotmail.com (C.L.); xue_bin04@163.com (B.X.); szq922990@126.com (Z.S.); jinminzh@126.com (M.J.); 2 State Key Laboratory of Molecular Developmental Biology, Institute of Genetics and Developmental Biology, Chinese Academy of Sciences, Beijing 100101, China; linyang@genetics.ac.cn

**Keywords:** internalization, logistic model, mechanical damage, nanoparticles

## Abstract

While nanoparticles exert bactericidal effects through the generation of reactive oxygen species (ROS), the processes of the internalization of and the direct physical damage caused by iron oxide nanoparticles are not completely clear. We hypothesize that direct physical or mechanical damage of the cell membrane and cytoplasmic integrity by nanoparticles is another major cause of bacterial death besides ROS. The aim of this study is to investigate the process of the internalization of iron oxide nanoparticles, and to evaluate the effect of direct physical or mechanical damage on bacterial cell growth and death. The results demonstrate that iron oxide nanoparticles not only inhibited *E. coli* cell growth, but also caused bacterial cell death. Iron oxide nanoparticles produced significantly elevated ROS levels in bacteria. Transmission electronic microscopy demonstrated that iron oxide nanoparticles were internalized into and condensed the cytoplasm. Strikingly, we observed that the internalized nanoparticles caused intracellular vacuole formation, instead of simply adsorbing thereon; and formed clusters on the bacterial surface and tore up the outer cell membrane to release cytoplasm. This is the first time that the exact process of the internalization of iron oxide nanoparticles has been observed. We speculate that the intracellular vacuole formation and direct physical or mechanical damage caused by the iron oxide nanoparticles caused the bactericidal effect, along with the effects of ROS.

## 1. Introduction

Nanomaterials, with their large specific surface area, high chemical reactivity, and biological activity, are rapidly growing in prominence in human health, biological processes, and environmental applications [1]. In particular, their great potential in advancing water and wastewater treatment to improve treatment efficiency may revolutionize conventional water treatment processes [2,3,4]. 

Iron oxide nanoparticles are a special class of metal oxide nanomaterials, with unique magnetic properties, high adsorbing properties, and superior bioactivities. Consequently, iron oxide nanoparticles have an extensive history of application in the field of environments and biomedical engineering, including their use as contrast agents for magnetic resonance imaging [5] and magnetic fluid hyperthermia [6], carriers for targeted drug delivery [7], magnetic separation of immune cells [8], tissue engineering applications [9], orthopedic implant infection prevention [10], arsenic (V) removal [11], environmental remediation [12], and wastewater treatment [13]. 

In addition, nanomaterials have great potential in the area of biological processes. Some nanomaterials have adverse effects on human health and the environment [14,15]. Indeed, some studies have demonstrated toxicological effects of nanoparticles on animals and fish [16], and human cell lines [17]. In the meantime, many kinds of nanoparticles, such as nanosilver, nano-alumina, and nano-titanium dioxide, have an antibacterial effect on many bacterial species [18,19,20]. Several recent studies have reported on the antimicrobial activity of nanoparticulate zero valent iron (nZVI), ferrous ion (Fe(II)), and magnetite nanoparticles (nMagnetite) [10,21,22,23].

Currently, it is commonly known that nanoparticles exert their biological effects through the generation of ROS [24,25], which induce apoptosis with no visible membrane damage [26,27,28] via internalization [29,30,31,32,33,34,35] and alteration of the bacterial cell membrane structure [16,17,18,24,25,36,37,38,39,40], thus leading to the accumulation of nanoparticles in the cell membrane and subsequent internalization [8,33,34,35]. However, the processes of this internalization and the direct physical damage caused by nanoparticles are not completely clear.

The inactivation of *E. coli* can also result from the oxidants generated inside and outside cells, as well as damage to the bacterial membrane induced by nZVI, Fe (II), and nMagnetite [10,21,22,23]. The notion that nanoiron oxide and other nanomaterials cause bacterial death through bacterial cell membrane damage and internalization has become the general consensus. However, no report has been made on how ferric oxide nanoparticles damage the bacterial membrane and become internalized.

In this study, we used the sensitive bacterial strain *E. coli* K12 to investigate the antibacterial and growth-inhibiting effects of ferric oxide nanoparticles, and to elucidate the process of the internalization of ferric oxide nanoparticles.

## 2. Results

### 2.1. Effect of Ferric Oxide Nanoparticles on Bacterial Culturability

**Figure 1:**
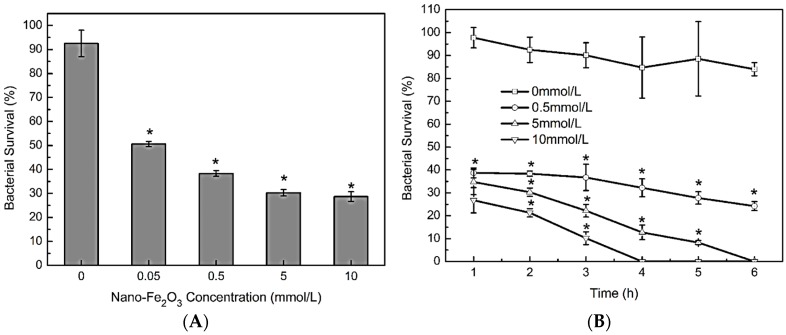
Culturabilityloss of *Escherichia coli* induced by iron oxide nanoparticle exposure in PBS. (**A**) *E. coli* MG1655 at 10^7^ colony forming units (CFU)/mL were exposed to 0, 0.05, 0.5, 5, or 10 mM iron oxide nanoparticles at pH 7.4 and 37 °C for 2 h.The presence of iron oxide nanoparticles significantly reduced the culturability of the bacteria (ANOVA, *p* < 0.05); significant differences between each concentration of the nanoparticles and the control (0 mM) were found with the Student–Newman–Keuls (SNK) test, * *p* < 0.05; (**B**) *E. coli* MG1655 at 10^7^ CFU/mL was exposed to 0.5 mM iron oxide nanoparticles for different time periods at pH 7.4 and 37 °C. With prolonged exposure to iron oxide nanoparticles, bacterial viability was significantly reduced (ANOVA, *p* < 0.05); significant differences between the iron oxide nanoparticle group and the control (0 mM) at each time point were tested by the SNK test, * *p* < 0.05.

Bacterial culturability experiments were conducted in PBS buffer, in which the bacteria were viable, but did not grow. The bacteria were first exposed to different concentrations of ferric oxide bulk particles diluted in PBS, and bacterial survival curves were plotted. No effect of the bulk particles on the culturability of *E. coli* MG1655 was observed (Figure S1). However, when the bacteria were exposed to different concentrations of the ferric oxide nanoparticles for 2 h, the bacteria died rapidly (Figure 1A). Even exposed to a low concentration of nanoparticles (0.05 mM) for 2 h, nearly half of the cells died. A significant dose effect of the ferric oxide nanoparticles was observed, and the survival numbers of the bacteria were significantly decreased as the concentration of ferric oxide nanoparticles and exposure time increased. Of note, concentrations of 5 and 10 mM of ferric oxide nanoparticles completely killed 10^7^ CFU/mL of the bacterial cells within 6 h or less (Figure 1B). These results demonstrate that ferric oxide nanoparticles affected bacterial culturability.

### 2.2. Growth Inhibition of E. coli by Ferric Oxide Nanoparticles

**Figure 2:**
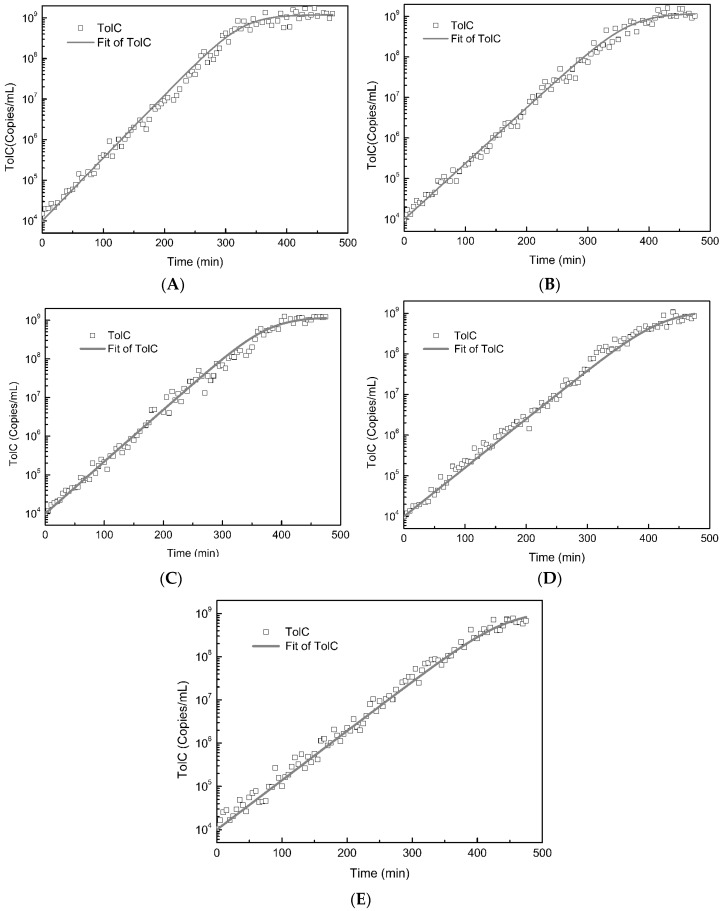
Bacterial growth curves according to qPCR of TolC and mathematical model fitting. (**A**) No exposure to iron oxide nanoparticles; *Ψ* = 0.03567 min^−1^, R^2^ = 0.89829; (**B**) Exposure to 0.05 mM iron oxide nanoparticles; *Ψ* = 0.03166 min^−1^, R^2^ = 0.92372; (**C**) Exposure to 0.5 mM iron oxide nanoparticles; *Ψ* = 0.03092 min^−1^, R^2^ = 0.96471; (**D**) Exposure to 5 mM iron oxide nanoparticles; *Ψ* = 0.02762 min^−1^, R^2^ = 0.93038; (**E**) Exposure to 10 mM iron oxide nanoparticles; *Ψ* = 0.0263 min^−1^, R^2^ = 0.93326.

The *E. coli* were grown in Luria-Bertani (LB) broth to an initial density 1 × 10^4^ CFU/mL, and the growth inhibition experiments were performed with the different concentrations of ferric oxide nanoparticles of 0, 0.05, 0.5, 5, and 10 mM.Bacteria growth was logistic in the absence of ferric oxide nanoparticles. The bacteria reached equilibrium with a bacterial density of about 1.2 × 10^9^ CFU/mL in 6 h (Figure 2A). However, when exposed to the different concentrations of ferric oxide nanoparticles, the bacterial growth rate decreased, and bacterial growth did not reach equilibrium in 6 h (Figure 2B–E), even when exposed to ferric oxide nanoparticles at 5 or 10 mM and incubated for 8 h (Figure 2D,E). To quantitatively evaluate the growth inhibition by ferric oxide nanoparticles, we fitted the growth curve data using a mathematical model, and calculated the growth rates with or without ferric oxide nanoparticles. The mathematical model fitted well to all of the growth curves, based on Quantitative PCR (qPCR) of the TolC locus (R^2^ > 0.898, Figure 2). Without nanoparticles being considered in the model, the growth rate was *Ψ* = 0.03587 min^−1^ based on the growth curve, which is similar to the growth rate of *E. coli* K12 W6 (0.035 min^−1^),as reported in a previous study [21]. Ferric oxide nanoparticles decreased the growth rates from 0.03587 min^−1^ to 0.0263 min^−1^, as the concentrations increased from 0 mM to 10 mM (Figure 2). There existed a significant dose effect relationship between bacterial growth inhibition and the concentrations of ferric oxide nanoparticles, i.e., the growth inhibition effect increased with increasing ferric oxide nanoparticle concentration. These results suggest that ferric oxide nanoparticles inhibited *E. coli* growth. 

### 2.3. Effects of Ferric Oxide Nanoparticles on Bacterial Morphology and Internalization of the Nanoparticles

**Figure 3:**
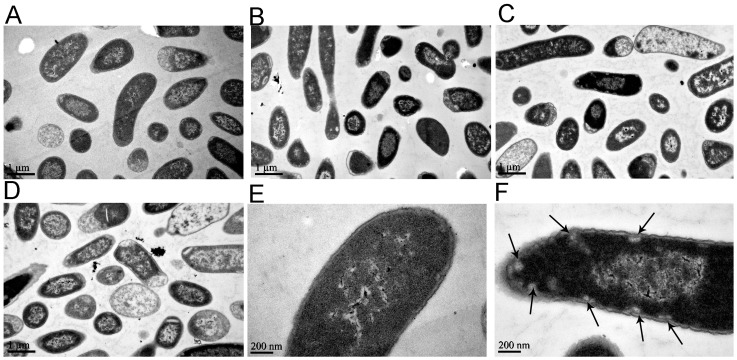
Transmission electronic microscopy of 10^7^ colony forming units (CFU)/mL of *E. coli* after exposure to iron oxide nanoparticles at 37 °C for 4 h. (**A**) No exposure to iron oxide nanoparticles in PBS; (**B**) Exposure to 0.05 mM iron oxide nanoparticles in PBS; (**C**) Exposure to 5 mM iron oxide nanoparticles in PBS; (**D**) Exposure to 10 mM iron oxide nanoparticles in PBS; (**E**) Partially magnified images of (**A**), showing that the cell membranes are distinct and the cytoplasm compact; (**F**) Partially magnified image of (**C**), showing vacuoles in cytoplasm as indicated by black arrows.

In order to understand why the *E. coli* were sensitive to the ferric oxide nanoparticles, the bacterial morphology and internalization of the nanoparticles were characterized by TEM. In the control group, the bacterial cell membranes were intact with clear cell borders, and compact and even cytoplasm (Figure 3A,E). The bacterial cells exposed to the different concentrations of ferric oxide nanoparticles were more deteriorated, with damage of the cell membrane and condensation of the cytoplasm as the concentration of nanoparticles increased (Figure 3B–D); in particular, many bacteria died (Figure 3C,D) at nanoparticle concentrations of 5 or 10 mM. Of note, many vacuoles close to membranes were observed in the cytoplasm of cells exposed to nanoparticles (Figure 3F), and some bacteria died without membrane damage (Figure 3C,D). 

**Figure 4:**
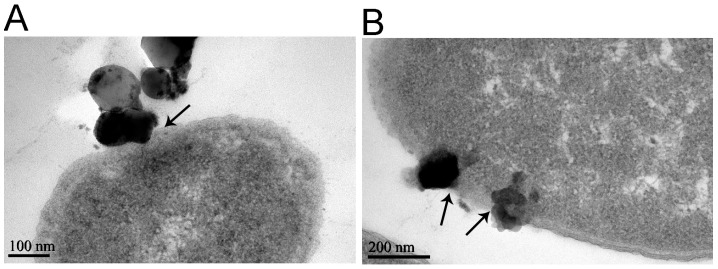

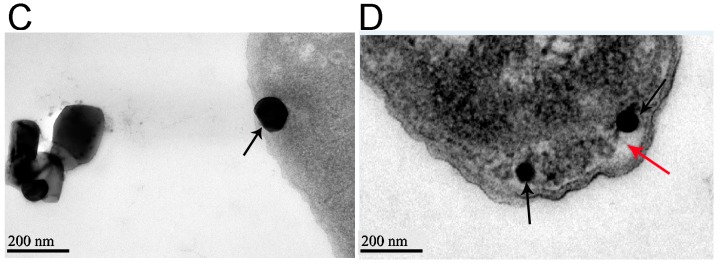
Transmission electron microscopy of the process of adsorption of iron oxide nanoparticles to *E. coli*. The bacteria, at 10^7^ colony forming units (CFU)/mL, were exposed to 0.5 mM iron oxide nanoparticles at 37 °C for 4 h in PBS. (**A**) Iron oxide nanoparticles approach the cell membrane and begin to invade, as indicated by the black arrow; (**B**) Iron oxide nanoparticles embed in the cell membrane and complete semi-penetrating, as indicated by black arrows; (**C**) Iron oxide nanoparticles are completely through the cell membrane, as indicated by the black arrow; (**D**) Iron oxide nanoparticles enter the cytoplasm, as indicated by black arrows; and vacuoles, indicated by red arrows, form around the nanoparticles in the cytoplasm.

**Figure 5:**
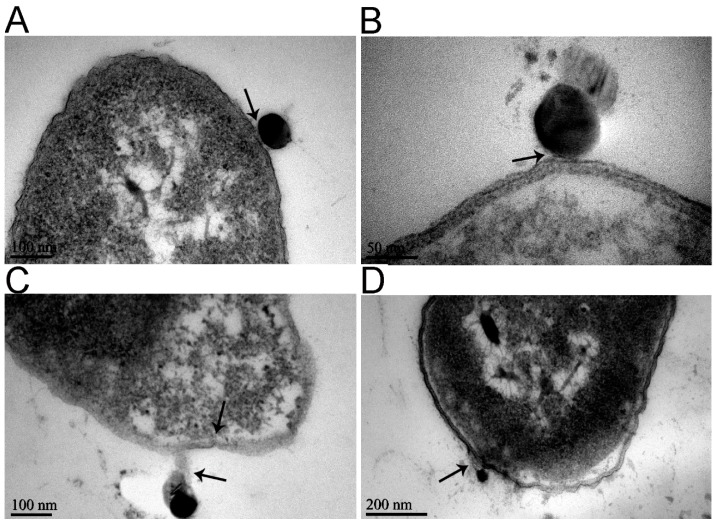
Transmission electron microscopy of the process of the destruction of *E. coli* cell membranes by iron oxide nanoparticles. The bacteria, at 10^7^ colony forming units (CFU)/mL, were exposed to 0.5 mM iron oxide nanoparticles at 37 °C for 4 h in PBS. (**A**) Iron oxide nanoparticles approach the cell membrane and adhere to the cell surface, as indicated by the arrow; (**B**) Iron oxide nanoparticles adhere to the cell’s outer membrane and tear the outer membrane to be uplifted, as indicated by the arrow; (**C**) Iron oxide nanoparticles adhere to the outer membrane closely to allow outer membrane release. The two regions indicated by black arrows were almost entirely coincident; (**D**) The cell membrane is broken and cytoplasm is released, as indicated by the black arrow.

It was also found that the internalization of the nanoparticles into the cell (Figure 4) damaged the cell membranes (Figure 5). The nanoparticles first approached the cell membrane and began to invade (Figure 4A), and then destroyed the membrane and embedded into it (Figure 4B). After completely penetrating through the cell membrane (Figure 4C), the ferric oxide nanoparticles entered the cytoplasm, causing vacuoles to form around these nanoparticles in the cytoplasm (Figure 4D). The process whereby the nanoparticles destroyed the membranes was as follows: the ferric oxide nanoparticles approached the cell and adhered to the cell surface (Figure 5A); adhered to the cell’s outer membrane and tore the outer membrane; uplifted the membranes (Figure 5B); adhered closely to the outer membrane and made it separate from the cell membrane (Figure 5C); and finally, broke the cell membrane, causing the release of cytoplasm (Figure 5D). The elemental analysis results showed that there were high levels of Fe in the regions around the highly dense particles in the bacteria (Figure S2), indicating that the highly dense particles were ferric oxide nanoparticles.

### 2.4. Effect of Ferric Oxide Nanoparticles on Intracellular ROS Level

**Figure 6:**
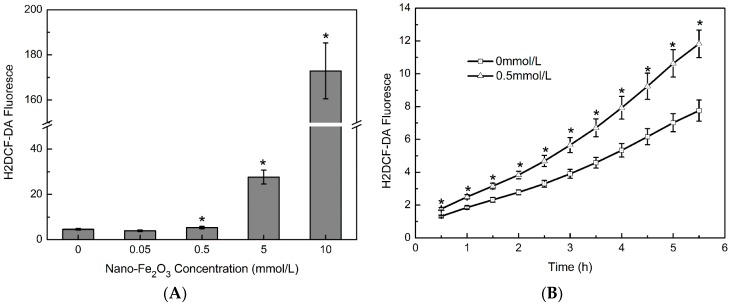
The intracellular level of reactive oxygen species. (**A**)The bacteria were exposed to 0, 0.05, 0.5, 5, or 10 mM iron oxide nanoparticles at 25 °C for 2 h in Luria-Bertani (LB) broth. Iron oxide nanoparticles significantly increased the fluorescence of H2DCF-DA (ANOVA, *p* < 0.05); significant differences between each concentration group and the control (0 mM) were found with the Student–Newman–Keuls (SNK) test, * *p* < 0.05; (**B**) The bacteria were exposed to 0.5 mM iron oxide nanoparticles for the different times indicated, at 25 °C in LB broth. With prolonged exposure to the iron oxide nanoparticles, the fluorescence of H2DCF-DA significantly increased (ANOVA, *p* < 0.05); significant differences between each of the iron oxide nanoparticle groups and the control (0 mM) at each time point were found with the SNK test, * *p* < 0.05.

To elucidate the mechanisms of ferric oxide nanoparticle toxicity to bacteria, we determined the effect of nanoparticle exposure on intracellular ROS levels using H2DCF-DA staining (Figure 6). After two hours of exposure to different concentrations of iron nanoparticles, a significant increase in ROS levels was induced; in particular, ROS levels in the 0.5, 5, and 10 mM ferric oxide nanoparticle-treated bacterial cells were significantly higher than those in the control group (Figure 6A). The intracellular ROS levels of each group increased with exposure time, and the ROS level in the 0.5 mM nanoparticle-treated cells was significantly higher than that of the control group at each time point (Figure 6B). These results indicated that the ferric oxide nanoparticles affected the oxidative stress-response systems of bacteria.

## 3. Discussion

In this study, we investigated the different stages of the internalization of nanoparticles, from their approach of the membrane to entry of the cytoplasm, for the first time (Figure 4); and we found that the direct physical damage from intracellular vacuole formation around nanoparticles and rupture of the cellular membrane may cause bacterial cell death, aside from the effects of ROS.

We first observed the antibacterial properties of ferric oxide nanoparticles in *E. coli*, although it has been reported that the chemically stable nanoparticles (*γ*-ferric oxide) have no apparent cytotoxicity to wild *E. coli* at 700 mg/L [21]. More importantly, we also demonstrated that the ferric oxide nanoparticles not only killed the bacteria in a non-nutritive culture (PBS), but also inhibited the bacteria growth in a nutritive culture (LB broth). The bactericidal effect was promoted with increased nanoparticle concentration and exposure time, even at very low concentrations, such as 0.05 mM (8 mg/L), which is much lower than that used in water or wastewater treatment [13,14]. 

Our results demonstrate that exposure to the ferric oxide nanoparticles increased cellular ROS levels (Figure 6), which may significantly reduce the ability of bacteria to survive and grow (Figure 1 and Figure 2), and caused some bacteria to die without cell membrane damage (Figure 3C,D). In fact, it is believed that iron-based nanoparticles, including magnetite nanoparticles and zerovalent iron nanoparticles, may generate ROS in bacterial cells due to the strong affinity of the nanoparticles for the bacterial cell membrane [21]. Nanomaterials can promote the production of endogenous ROS inside the bacterial cells [41]. Under unfavorable environments, such as hypoxia or in the presence of toxins, oxidative stress occurs, and endogenous ROS accumulation can damage cellular constituents and disrupt cell functions, and thus being responsible for strong bactericidal activity [26,41]. Bacterial cell death without visible membrane damage may be attributed to this ROS-induced apoptosis of the cell [26,27,28].

Through TEM, we found that although the extent of internalization increased, few ferric oxide nanoparticles adsorbed and formed clusters on the bacterial surface, even at high concentration of the ferric oxide nanoparticles (Figure S3). We also observed damaged membranes and condensed cytoplasm of the bacteria (Figure 3). These results differ from a previous study, where most of the TiO_2_ and Al_2_O_3_ nanoparticles and single- and multiwalled carbon nanotubes adsorbed onto the bacterial surface [20]. In addition to the internalization of some nanoparticles, we found that ferric oxide nanoparticles adhered to the cell’s outer membrane and tore up the membrane, leading to the cell membrane swelling and rupturing to release cytoplasm (Figure 5). We postulate that the impairment of cell membrane and cytoplasmic integrity as induced by the ferric oxide nanoparticles may be another major cause of bacterial death.

*E. coli*, being a Gram-negative bacteria, possesses a cell wall that consists of an outer membrane containing lipopolysaccharides, a periplasmic space with a peptidoglycan layer, and an inner cytoplasmic membrane. We found that nanoparticles invaded and embedded into the cell membrane. The process of iron oxide nanoparticles crossing these two lipid bilayers may be similar to that of nanoparticles crossing plasma membranes, as reported by some researchers [42,43]. Furthermore, cytoplasmic nanoparticles induced vacuole formation around the nanoparticles, leading to damage of the cell membrane structure and cytoplasm. However, while many metal oxide nanoparticles change the bacterial cell membrane structure [18,19,20,21,24,25,36,37,38,39,40,41] and increase membrane permeability, leading to the accumulation of nanoparticles in the cell membrane and subsequent internalization [21,33,34,35], nanoparticles had not been found to embed into the cell membrane or cause vacuole formation before. We speculate that intracellular vacuole formation is another type of physical damage caused by nanoparticles. This is the first observation of its kind, to the best of our knowledge, and may be a new pathway for ferric oxide nanoparticles to cause bacterial death without cell membrane damage (Figure 3F and Figure 4D). The new pathway raises the question as to how much ROS contribute to a bactericidal effect. 

In conclusion, we postulate that intracellular vacuole formation and rupture of the cell membrane may cause direct physical damage, which in turn contributes to bacterial cell death, along with the effects of ROS. 

## 4. Materials and Methods

### 4.1. Ferric Oxide Nanoparticles

The iron(III) oxide nanoparticle dispersion (Product No. 720712) purchased from Sigma-Aldrich (St. Louis, MO, USA) were measured as having primary particle diameter smaller than 110 nm, by dynamic light scattering, and average particle diameter smaller than 30 nm, by aerodynamic particle sizer. The crystal type of ferric oxide nanoparticles was tested using X-ray photoelectron spectroscopy (XPS, Escalab 250Xi, Thermo Fisher Scientific, Waltham, MA, USA). The results (Figure S4) showed that the crystal type of the ferric oxide nanoparticles was alpha. A series of stock solutions of 0.5, 5, 50, and 100 mM ferric oxide nanoparticles suspended in ultrapure water was prepared. To avoid aggregation, the suspensions were sonicated in sealed sterile tubes for 30 min before being added to bacterial cells.Another ferric oxide powder (Product No. 310050), with particle size <5 μm, purchased from the same company was resuspended with ultrapure water to the same concentrations as the nanoparticles above, and used for bulk particle controls. 

### 4.2. Bacteria

The Gram-negative *Escherichia coli* K12 MG1655 (ATCC 47076, Rockville, MD, USA) was used as the model bacteria. The bacteria were inoculated into Luria-Bertani (LB) media containing 5 g/L of yeast extract, 10 g/L of bactotryptone, and 5 g/L of NaCl, and grown at 37 °C overnight with agitation. Then, the bacteria were harvested by centrifugation at 4000× *g* for 5 min, washed three times with phosphate buffered solution (PBS, pH 7.4), and resuspended in PBS to derive a bacterial stock solution at a density of 10^8^ colony forming units (CFU)/mL.

### 4.3. Exposure of E.coli to Ferric Oxide Nanoparticles in PBS

Ferric oxide nanoparticle solution (0.1 mL) or bulk particle stock solution was added to 9.9 mL of bacterial suspension (1 × 10^7^ CFU/mL), diluted from the bacterial stock solution with PBS to derive final ferric oxide solutions of 0.05, 0.5, 5, or 10 mM. Meanwhile, bacterial controls were prepared similarly, but without ferric oxide. All the bacterial suspensions were incubated at room temperature with gentle stirring. After the designed exposure time, the live bacteria numbers were determined by counting the numbers of CFUs on solid LB agar plates, which were incubated at 37 °C for 16–18 h. The culturability loss of the bacteria was plotted, either against different concentrations of the ferric oxide nanoparticles for 2 h, or different time periods at 0.5 mM ferric oxide nanoparticles. The bacterial survival rate was expressed as N/N_0_ (%), where N and N_0_ are the remaining and initial numbers of live bacteria (CFU/mL), respectively.

### 4.4. Exposure of E.coli to Ferric Oxide Nanoparticles in LB Broth

Bacterial stock solutions (each 10 μL) were inoculated into 100 mL LB broth in 250 mL flasks at final concentration 1 × 10^4^ CFU/mL. Then, the ferric oxide nanoparticle stock suspension or sterile distilled water was mixed with the bacteria in the flasks with vortexing, to derive ferric oxide nanoparticle stock solutions at 0, 0.05, 0.5, 5, and 10 mM. The flasks were shaken in a thermostat shaker at 37 °C and 120 rpm. A 10 μL aliquot was taken every 5 min without pausing the shaking. The 10 μL aliquot was diluted into 990 μL sterile distilled wate rand placed in a boiling water bath for 10 min, to denature potential degradation enzymes and preserve the bacterial cell number. All the aliquots were stored at −20 °C until qPCR assays were conducted. 

The qPCR assays were used to quantify bacterial amount at every time point, using the genetic locus TolC of the bacteria [44] and Power SYBR^®^ Green Master Mix (Applied Biosystems, Foster, CA, USA) in an Applied Biosystems 7300 (Applied Biosystems, Foster, CA, USA). Lysed and frozen aliquots of the bacteria (2 μL) were used as templates for the qPCR reactions (20 μL). The primer sequences were as follows: TolC-F: TTGATCGCGCTAAATACTGCT; TolC-R: AGGCGTGCTTGCTGATAAAC. The qPCR conditions were as follows: an initial denaturation at 95 °C for 30 s; 40 cycles of 95 °C for 5 s; 60 °C for 60 s. We set the following mathematical model to analyze the effect of ferric oxide nanoparticles on bacterial growth. As batched bacterial cell cultures were assayed, we assumed a logistic bacterial growth, as described previously [44,45]. The logistic model states that the number of cells in a state as a function of time (*N*′) is the product of the growth rate (*ψ*), the number of cells currently in that state (*N*), and the saturation limit of the media (*K*).

(1) $$N\prime =\psi N(1-\frac{N}{K})$$

We used the solve function of MATLAB (Ver. 8.1.604 (R2013a), The MathWorks, Natick, MA, USA) to solve Equation (1), and obtained the analytical solution of *N* on time (*t*) as follows:

(2) $$N(t)=\frac{K}{\frac{K\times e^{\psi \times t}}{A}-e^{-\psi \times t}+1}$$

We set the initial conditions of *N* as A copies/mL (cfu/mL). Using the nonlinear fitting tool of OriginPro 8 SR2 (Ver. 80891 (B891), OriginLab Corporation, Northampton, MA, USA), we calculated the parameters *ψ*. The carrying capacity, *K*, and initial conditions (or initial bacterial density), A, were approximately 1.2 × 10^9^ copies/mL (cfu/mL) and 1 × 10^4^ copies/mL (cfu/mL), respectively. 

### 4.5. Transmission Electron Microscopy of Bacteria Exposed to Ferric Oxide Nanoparticles

After the exposure, the bacteria were fixed at 4 °C with 2.5% glutaraldehyde fixative and 2% OsO_4_ in anhydrous acetone that contained 8% dimethoxypropane, for 2 h and 2 days, respectively, and dehydrated using an ethanol gradient. Then, the bacteria were treated with propylene oxide and embedded in Epon812 resin (Shell Chemical, Stanlow, UK). Ultrathin sections (50 nm) were cut and counterstained with Reynold’s and uranyl acetate, and observed under a JEM-1400 transmission electronic microscope. The elements in the bacterial slice were analysed by high-resolution TEM (FEI TECNAI G2F20, Hillsboro, OR, USA) with an X-ray energy dispersive analysis system (GENESIS, Mahwah, NJ, USA). 

### 4.6. ROS Assays

The cellular level of ROS assay was as described previously [20,21], with minor modification. Aliquots of the overnight cultured bacteria were centrifuged at 4000× *g* for 5 min. The pellet was then resuspended in LB broth containing 10 μM H_2_DCF-DA (dichlorodihydrofluorescein diacetate, Invitrogen, Carlsbad, CA, USA) and incubated in 37 °C for 30 min. After the LB broth was removed, the bacterial cells were inoculated into prewarmed LB broth containing ferric oxide nanoparticles at designed concentrations, and placed into a black 96-well plate. The incubation was allowed to proceed for designated times at room temperature in the dark. Then, the fluorescence intensity of cell/nanoparticle suspensions was measured using Fluoroskcan Ascent FL (Thermo Scientific, Waltham, MA, USA) at excitation and emission wavelengths of 485 nm and 538 nm, respectively.

### 4.7. Statistical Analysis

All experiments were repeated at least three times independently. Data are expressed as means ± standard deviation (SD). All data were analysed with SPSS for Windows version 17.0 (SPSS Inc., Chicago, IL, USA). *p*-Values less than 0.05 were considered to be statistically significant.

## Supplementary Materials

The following are available online. Figure S1: The XPS of iron oxide nanoparticles. Figure S2: Viability loss of *Escherichia coli* induced by iron oxide bulk particle exposure in PBS. Figure S3: The composition of chemical elements of highly dense particles in bacteria. Figure S4: Iron oxide nanoparticles adsorbed on bacterial surfaces.
