# Supplementary material for: The Detailed Bactericidal Process of Ferric Oxide Nanoparticles on E. coli
Source: Molecules. 2018 Mar 8;23(3):606. doi: 10.3390/molecules23030606 (PMC6017908; doi:10.3390/molecules23030606)
Supplement: Supplementary file 1 [file molecules-23-00606-s001.pdf]

Supplementary Materials

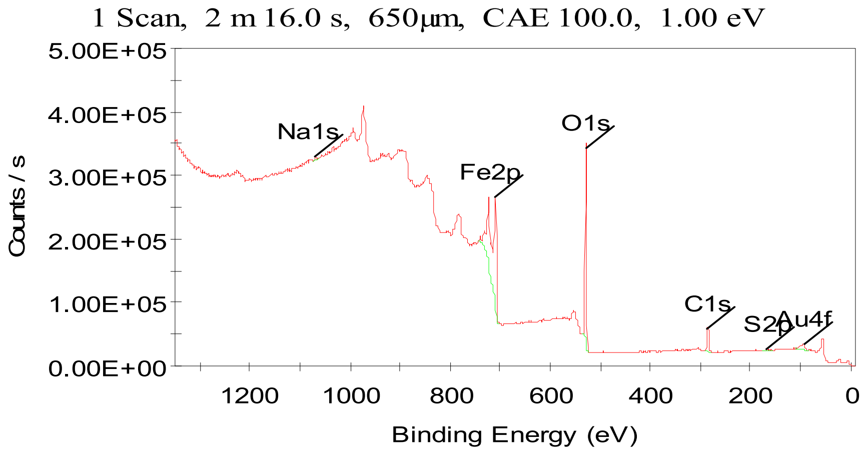

Figure S1 The XPS of iron oxide nanoparticles

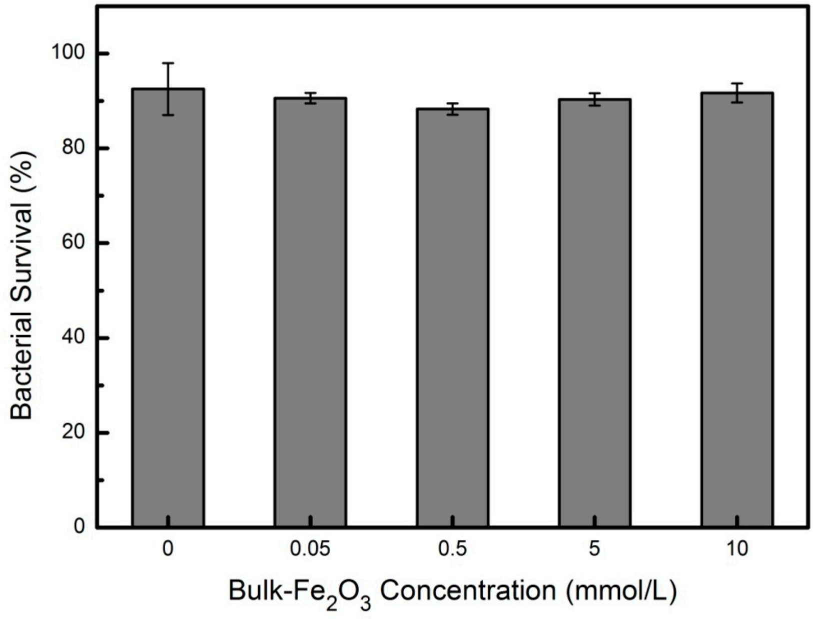

Figure S2. Viability loss of *Escherichia coli* induced by iron oxide bulk particles exposure in PBS. *E. coli* MG1655 at 10^7 colony formation unit (CFU)/mL were exposed to 0, 0.05, 0.5, 5, or 10 mM iron oxide bulk particles at pH 7.4 and 37°C for 2 h. The presence of iron oxide bulk particles did not significantly reduced the viability of the bacteria (ANOVA, P >0.05); no significant differences between each

concentration of the nanoparticles and the control (0mmol/L) were found with the Student-Newman-Keuls (S-N-K) test.

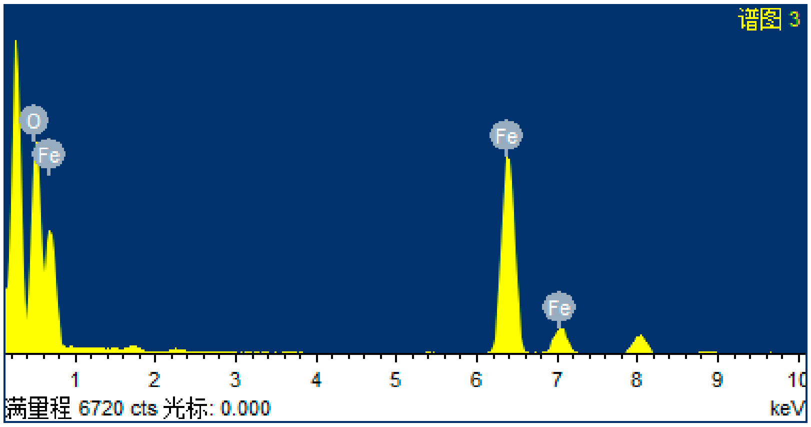

Figure S3. The composition of chemical elements of highly dense particles in bacteria. Possible neglected peaks: 1.739, 2.298, 5.440, 8.041, 8.895, 9.697 and 11.431 keV which originated from the copper net, whereas lead, arsenic, dye liquid and bacteria.

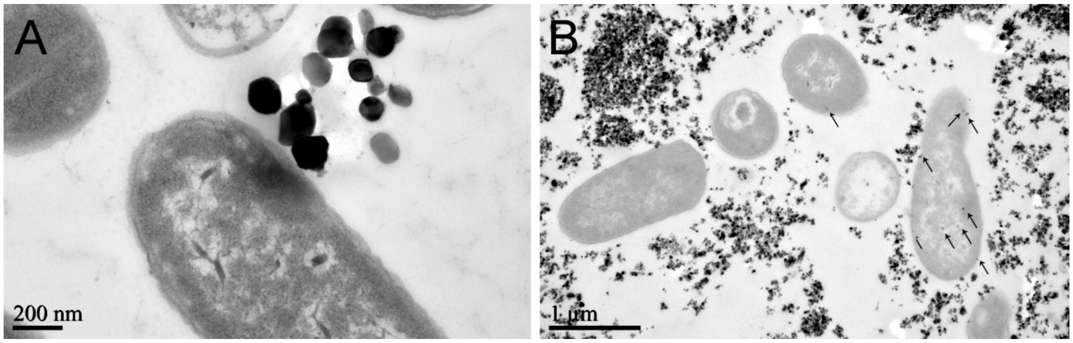

Figure S4. Iron oxide nanoparticles adsorbed on bacterial surfaces. A, large size graph; B, small size graph, arrows indicates the nanoparticles internalization.
